# Increased arginine, lysine, and methionine levels can improve the performance, gut integrity and immune status of turkeys but the effect is interactive and depends on challenge conditions

**DOI:** 10.1186/s13567-022-01080-7

**Published:** 2022-07-26

**Authors:** Paweł Konieczka, Bartłomiej Tykałowski, Katarzyna Ognik, Misza Kinsner, Dominika Szkopek, Maciej Wójcik, Dariusz Mikulski, Jan Jankowski

**Affiliations:** 1 Department of Poultry Science, University of Warmia and Mazury in Olsztyn, 10-719 Olsztyn, Poland; 2 Department of Poultry Diseases, Faculty of Veterinary Medicine, University of Warmia and Mazury in Olsztyn, 10‑719 Olsztyn, Poland; 3 Department of Biochemistry and Toxicology, University of Life Sciences, 20-950 Lublin, Poland; 4 Department of Animal Nutrition, The Kielanowski Institute of Animal Physiology and Nutrition, Polish Academy of Sciences, Instytucka 3, 05-110 Jabłonna, Poland

**Keywords:** Turkey, essential amino acids, gut health, immune response, redox balance

## Abstract

Arginine (Arg), lysine (Lys), and methionine (Met) can be used to support the health status of turkeys. The present study investigated selected performance, gut integrity, and immunological parameters in turkeys reared in optimal or challenge conditions. The experiment lasted for 28 days, and it had a completely randomized 2 × 3 factorial design with two levels of dietary Arg, Lys and Met (high or low) and challenge with *Clostridium perfringens* (*C. perfringens*), *Escherichia coli* lipopolysaccharide (LPS) or no challenge (placebo). Increased dietary levels of Arg, Lys and Met had a beneficial effect on turkey performance and immunological parameters, and it improved selected indicators responsible for maintaining gut integrity in different challenge conditions. Under optimal conditions (with no challenge), high ArgLysMet diets did not compromise bird performance and they improved selected performance parameters in challenged birds. The immune system of turkeys was not excessively stimulated by high ArgLysMet diets, which did not disrupt the redox balance and had no negative effect on gut integrity. High ArgLysMet diets increased the expression levels of selected genes encoding nutrient transporters and tight junction proteins. However, the influence exerted by different dietary inclusion levels of Arg, Lys and Met on gut integrity was largely determined by the stressor (*C. perfringens* vs. LPS). Further studies are required to investigate the role of Arg, Lys and Met levels in the diet on the immune response, gut function and performance of turkeys in different challenge conditions.

**Supplementary Information:**

The online version contains supplementary material available at 10.1186/s13567-022-01080-7.

## Introduction

Measures should be taken to improve the health status of birds (including turkeys) raised under intensive farming systems. An improvement in gastrointestinal tract (GIT) function is an important consideration. The GIT ecosystem and the resident microbiota constitute the first line of defense against pathogens and are the key components of innate and adaptive immunity [1]. Therefore, GIT function and integrity should be enhanced to stimulate defense mechanisms in birds as part of non-specific prevention [2]. Intestinal health is critical for maximizing growth performance and production efficiency in turkeys. When gut homeostasis is disrupted by pathogens, nutrient digestion and absorption are altered since priorities are shifted from maintaining regular physiological processes to fighting off the pathogens [3].

Essential amino acids (EAAs), including arginine (Arg), lysine (Lys) and methionine (Met), play a key role in supporting gastrointestinal function and the gut-associated immune system. Experiments performed on chickens demonstrated that increased dietary levels of EAAs stimulated local immunity [4], and contributed to reducing intestinal mucosa atrophy [5] and maintaining intestinal microbiota diversity under both optimal and stress conditions [6]. However, the majority of studies investigating the regulatory role of EAAs involved animal models other than turkeys [7]. Due to considerable differences in metabolism and EAA requirements resulting from different growth rates, data cannot be directly extrapolated from other poultry species to turkeys. Our previous studies [8, 9] and the findings of other authors [10] point to numerous interactions in host responses depending on the proportions of individual EAAs in the diet, birds’ age and the applied stressors. Moreover, turkey diets are formulated based on the nutritional recommendations of two companies [11, 12] that differ in the dietary rates and ratios of Arg, Lys and Met, and are aimed at optimizing growth performance rather than supporting GIT and immune functions. Therefore, the maintenance of optimal (high or low) proportions of Arg and Lys in the diet seems particularly interesting. Both AAs are structurally similar, which suggests that the differences in bird responses to their dietary content may result from varying Arg:Lys ratios in the diet. Kidd and Kerr [13] found that an increase in the dietary Arg:Lys ratio exerted the greatest effect on the body weight (BW) and body weight gain (BWG) of turkeys at 8–20 and 20 weeks of age, respectively. However, there is no information on the consequences of increasing the dietary inclusion levels of not only Arg and Lys but also Met. Oso et al. [14] demonstrated that Arg supplementation (increased to dietary Met level) led to a linear improvement in nutrient digestibility in 84-day-old turkeys and increased nutrient absorption, as indicated by increased intestinal villus height. Waldroup et al. [15] reported that increasing the Arg:Met ratio did not improve performance when Lys levels were adequate.

In view of the above, the aim of this study was to determine the effect of increased dietary Arg, Lys and Met levels on performance, GIT and immunological parameters in young turkeys reared under optimal conditions or exposed to various stressors. We hypothesized that increased levels of Arg, Lys and Met, relative to those recommended by NRC [12], would be effective in maintaining or improve bird performance by supporting gut integrity and immune function in challenge conditions.

## Materials and methods

### Ethical statement

The study protocol was approved by the Local Ethics Committee (University of Warmia and Mazury, Olsztyn, Poland) resolution No. 57/2020 of 21 October 2020, and the animals were cared for under guidelines comparable to those laid down by EU Directive 2010/63/EU.

### Birds and housing

The experiment was performed on female Hybrid Converter turkeys purchased from the Grelavi Hatchery in Kętrzyn. One-day-old poults (a total of 192 birds) were randomly assigned to 48 cages (replicates) in the housing facility. Each of six group consisted eight replicates with 4 birds per replication. The experiment had a completely randomized design with six groups of eight replicate cages each, with four birds per cage. Replicates (cages) in groups were uniformly (homogeneously) distributed in the building. The microclimate in the housing facility was controlled automatically, the conditions were adjusted to the birds’ age, and were consistent with the recommendations of Hybrid Turkeys [16]. All birds were housed under identical conditions. Throughout the experiment, all birds had unlimited access to feed and water.

### Diets and experimental design

The experimental design is presented in Additional file 1. During the 28-day experiment, the birds were fed ad libitum isocaloric diets, which met or exceeded their nutrient requirements according to the nutrient guidelines for turkeys [16]. The experiment had a completely randomized 2 × 3 factorial design with two levels of dietary Lys, Arg and Met (high or low) and challenge with (i) *Clostridium perfringens* (*C. perfringens*), (ii) *Escherichia coli* lipopolysaccharide (LPS) or (iii) no challenge (placebo). Low ArgLysMet diets contained 16 g Lys per kg of diet, 90% Arg relative to Lys content and 30% Met relative to Lys content, according to NRC guidelines [12]. High ArgLysMet diets contained 18 g Lys per kg of diet, 110% Arg relative to Lys content and 45% Met relative to Lys content (Additional file 2). The content of AAs (Lys, Arg and Met) was analytically determined in the basal diet, and then the adequate amounts of AAs were added to reach their respective target levels in diets. The diets were offered as crumbles. Turkeys were divided into six groups: birds fed diets with low or high levels of Arg, Lys and Met (T1 and T2, respectively), birds fed the above diets and challenged with *C. perfringens* (T3 and T4, respectively), birds fed the above diets and challenged with LPS (T5 and T6, respectively).

### Challenge

At 25, 26 and 27 days of age, group T3 and group T4 birds were challenged with *C. perfringens* in accordance with the procedure developed in our laboratory [17]. Inoculum (1 mL) containing *C. perfringens* type A strain 56 in the amount of 2.3 × 10^7^ CFU (day 15) and 4.5 × 10^7^ CFU (day 16) was obtained by overnight incubation at 37 °C in brain heart infusion broth (Sigma Aldrich). The bacteria were administered directly into the crop with the use of a cannula. The amount of bacteria in the inoculum was tested analytically, according to Standard ISO 7937:2005 [18], in a veterinary laboratory (Avipoint, Olsztyn, Poland). The degree of intestinal mucosa damage was evaluated by a veterinarian (a poultry disease specialist) based on anatomopathological changes. A post-mortem examination revealed the absence of typical lesions that accompany clinical acute necrotic enteritis (NE) in turkeys [19, 20]. On the same days (25, 26 and 27 days of age), group T5 and group T6 birds were challenged with LPS in accordance with the previously described protocol [21]. Before the LPS challenge, birds were weighed individually, and LPS was administered (*Escherichia coli* serotype O55:B5; Sigma Chemical, St. Louis, MO, USA) at 250 μg/kg BW. Before administration, LPS was dissolved in sterile 0.9% NaCl solution (0.5 mg/mL). The intestines of turkeys challenged with LPS were subjected to an anatomopathological examination by a veterinarian (a poultry disease specialist).

### Gut permeability test

A gut permeability test was performed in turkeys aged 28 days. Before the test, turkeys were weighed, and eight birds per group were administered fluorescein-5-isothiocyanate dextran (FITC-d, Sigma-Aldrich) at 4.17 µg/kg BW directly into the crop with the use of a cannula. Two and a half hours after the administration of FITC-d, 0.5 mL blood samples were collected from the wing vein to determine FITC-d concentrations with a fluorimeter [22].

### Evaluation of turkey performance

The BW of birds were recorded and calculated on a cage basis. The feed conversion ratio (FCR; kg of feed/kg BWG) for the experimental period was calculated on a pen basis from BWG and feed consumption. Mortality rates and causes were recorded daily, and the weights of dead birds were used to adjust the average FCR.

### Sample collection

At 28 days of age, blood samples were collected from the wing vein of eight birds per group (different than those used in the gut permeability test) into tubes containing EDTA K2 for flow cytometry analyses, lithium heparin for biochemical and genetic analyses or clot activator for serological analyses. Blood samples for cytometric analyses were directly used for the isolation of mononuclear cells. The remaining samples were centrifuged for 15 min at 380 × *g* and 4 °C (plasma) or 10 min at 1000 × *g* and 4 °C (serum), and the resulting plasma and serum were stored at − 20 °C until analysis. Birds were sacrificed by decapitation after electrical stunning, and the abdominal cavity was opened for the collection of jejunum (middle-jejunum) tissues, liver and spleen samples.

### Laboratory analyses

Mononuclear cells were isolated from the blood and spleen in accordance with the protocol developed by Koncicki et al. [23]. The cells were counted, and their viability was evaluated using the Vi-Cell XR cell counter (Beckman Coulter, USA). The percentages of CD4^+^ and CD8α^+^ T cell and IgM^+^ B cell subpopulations in blood and the spleen were determined as described by Kubińska et al. [24]. Briefly; viable mononuclear cells (1 × 10^6^) were stained with FITC-conjugated Mouse Anti Chicken CD4 clone 2-35 (Bio-Rad, UK) and PE-conjugated Mouse Anti Chicken CD8α clone 11-39 (Bio-Rad, UK) or with FITC-conjugated Goat Anti Chicken IgM polyclonal IgG (Bio-Rad, UK). Data were acquired using a FACSCanto II digital flow cytometer (BD, USA) in the FACSDiva 8.0 environment (BD, USA). The immunophenotype and percentages of subpopulations of CD4^+^, CD8α^+^, CD4^+^CD8α^+^ double positive cells and B lymphocytes (IgM^+^) were analyzed using FlowJo V10 software (BD, USA). A fluorescence minus one (FMO) controls for all fluorochromes was used to determine the cut-off point between background fluorescence and positive populations. The cytometer setup and tracking beads (CST, BD, USA) were used to initialize photomultiplier tubes settings. Unstained and single-stained control cells for each fluorochrome were prepared and used to set up flow cytometry compensation. A gating strategy using a spleen sample as an example is shown in Additional file 3.

DNA was isolated from the intestinal wall using QIAGEN kits. Epigenetic changes in the blood and intestinal wall of turkeys were determined by analyzing global DNA methylation (methylome) with the use of Sigma Aldrich diagnostic kits. The levels of 8-hydroxydeoxyguanosine (8-OHdG), endonuclease 1 (APE-1) and oxoguanine glycosylase (OGG1) in the blood and intestinal wall of turkeys were determined using OxiSelect diagnostic kits (Cell Biolabs, Inc., San Diego, USA). OxiSelect diagnostic kits (Cell Biolabs, Inc., San Diego, USA) were also used to determine protein carbonyl (PC) and 3-nitrotyrosine (3-NT) derivatives as an indicator of the oxidation of AA residues. The levels of caspase 3 (Casp-3) and caspase 8 (Casp-8) were determined in the blood plasma and intestinal wall of turkeys using an ELISA kit (Cell Biolabs, Inc. San Diego, USA). The plasma levels of C-reactive protein (CRP) were determined in an ELISA reader using assays from Elabscience Biotechnology Co., Ltd. (Houston, Texas, USA). The levels of ceruloplasmin (Cp) in the plasma and jejunum of turkeys were determined using a Ceruloplasmin ELISA kit (Biomatik, Delaware, USA). The levels of total serum globulins and immunoglobulins IgA and IgY, tumor necrosis factor alpha (TNF-α), and interleukin 6 (IL-6) were determined in an ELISA reader using assays from Elabscience Biotechnology Co., Ltd. (Houston, Texas, USA). Anti-ORT IgY serum titers were determined using a commercial immunoenzymatic ELISA kit (IDEXX Laboratories, USA) according to the manufacturer’s recommendations. The ELISA assay was performed using an epMotion 5075 LH automated pipetting system (Eppendorf), an Elx405 washer, an Elx800 absorbance microplate reader (BioTek, USA) and the KBF 115 constant climate chamber (Binder, Tuttlingen, Germany).

### Analysis of mRNA expression levels

The mRNA expression levels of genes were quantified in ileum samples collected from birds at 21 days of age. Quantitative real-time PCR analysis was performed according to a previously described method with some modifications [25]. Briefly, total mRNA from the collected tissues was isolated using the Total RNA Mini Kit (A&A Biotechnology, Gdynia, Poland) according to the manufacturer's protocol. The yield of isolated RNA was assessed spectrophotometrically (Nanodrop, NanoDrop Technologies, Wilmington, DE) and integrity was assessed electrophoretically by separation on 1.5% agarose gel containing ethidium bromide. To synthesize complementary cDNA, 1000 ng/mL mRNA from selected tissues in a total volume of 20 μL was retrotranscribed using the Maxima First Strand cDNA Synthesis Kit for RT-qPCR, with ds DNase (ThermoFisher Scientific, Warsaw, Poland) according to the manufacturer’s instructions. Turkey (*Gallopavo meleagridis*) specific primers used for housekeeping and test gene expression determination (GLUT 1: Glucose transporter-1, GLUT 2: Glucose transporter-2, PEPT 1: Peptide transporter-1, PEPT 2: Peptide transporter-2, ASCT 1: Alanine, serine, cysteine, and threonine transporter, ZO 1: Zonula occludens 1, OCCL: Occludin, BoAT: Solute carrier family 6, member 19, SI: Sucrase isomaltase, EAAT 3: Excitatory amino acid transporter 3, CCK 1: Cholecystokinin type 1 receptor, CAT 1: Cationic amino acid transporter-1, CCK: Cholecystokinin) (Additional file 4) were designed using the Nacional Library of Medicine, National Center for Biotechnology Information (NCBI) (Bethesda, MD, USA) primer designing tool and synthesized by Sigma-Aldrich (Poland). Real-time qPCR was performed using 5 × FIREPol EvaGreen qPCR Mix Plus (no ROX; Solis BioDyne, Tartu, Estonia) in a total volume of 15 μL containing 3 μL Master Mix, 9 μL RNAse-free H2O, 2 × 0.5 μL primers (0.5 mM), and 2 μL cDNA template. Amplification was performed using a Rotor Gene 6000 thermocycler (Corbett Research, Mortlake, Australia) according to the following PCR protocol: one cycle at 95 °C for 15 min (enzyme activation); 35 cycles at 95 °C for 5 s (denaturation), 60 °C for 25 s (annealing), and 72 °C for 15 s (elongation); followed by one cycle at 72 °C for 7 min (product stabilization). Melting curve analysis was performed at 70–95 °C in 0.5 °C intervals. Negative controls without the cDNA template were included in each reaction. The real-time qPCR reaction for each cDNA sample was performed twice in duplicate. The identity of the PCR products was confirmed by direct sequencing. Relative gene expression was calculated using the comparative quantification option of Rotor Gene 6000 1.7 software (Qiagen GmbH, Hilden, Germany) and determined using the Relative Expression Software Tool based on the PCR efficiency correction algorithm. Phosphoglycerate Kinase 1 (PGK 1), Transferrin Receptor (TFRC) and Ribosomal Protein (RPS 7) genes (Additional file 4) were tested as host genes using NormFinder software. The results are presented as the relative expression of a target gene vs. a housekeeping gene and relative gene expression for a selected group of birds.

### Statistical analysis

The data were subjected to 2-way ANOVA to examine the following effects: (a) main effect of two levels (low or high) of dietary ArgLysMet; (b) main effect of challenge with *C. perfringens*, or with LPS, or with no challenge (placebo); and (c) interaction between dietary ArgLysMet level and challenge factor. All data were analyzed using the GLM procedure of STATISTICA software version 12. When a significant interaction effect was noted, Tukey’s test was used to determine differences between the experimental factors. Data variability was expressed as pooled standard errors of the mean (SEM), and *P* < 0.05 was considered statistically significant.

## Results

### Performance response to dietary treatments

The growth performance of turkeys before the challenge (days 0–25), during the challenge (days 25–28) and throughout the experiment (days 0–28) is presented in Tables 1 and 2. High and low dietary rates of AAs significantly affected selected performance parameters in turkeys. High ArgLysMet diets increased BW at 25 (*P* = 0.02) and 28 (*P* = 0.024) days of age, increased BWG on days 0–25 (*P* = 0.03) and increased BW during the entire experiment (days 0–28, *P* = 0.02). Experimental challenge with *C. perfringens* or *E. coli* LPS had no significant effect on the growth performance of turkeys (*P* > 0.05). Neither high or low dietary rates of AAs nor applied challenge significantly affected Fint or FCR in all investigated periods (*P* > 0.05).

**Table 1:** **Body weight (BW) and feed intake (Fint) of turkeys calculated for pre-, during-, and post-challenge periods**

Parameter		N	BW day 0, g	BW day 25, kg	BW day 28, kg	Fint days 0–25, kg	Fint days 25–28, kg	Fint days 0–28, kg
Diet^1^								
Low ArgLysMet		24	60.11	1.148^b^	1.337^b^	1.708	0.379	2.087
High ArgLysMet		24	60.1	1.207^a^	1.388^a^	1.757	0.423	2.18
Challenge^2^								
No		16	60.33	1.165	1.383	1.742	0.408	2.149
*C. perfringens*		16	59.83	1.178	1.349	1.702	0.391	2.093
LPS		16	60.15	1.19	1.354	1.755	0.404	2.159
Treatments								
Low ArgLysMet	No	8	60.42	1.154	1.355	1.71	0.372	2.082
Low ArgLysMet	*C. perfringens*	8	60	1.138	1.332	1.691	0.38	2.071
Low ArgLysMet	LPS	8	59.91	1.153	1.322	1.724	0.386	2.11
High ArgLysMet	No	8	60.23	1.175	1.411	1.773	0.444	2.217
High ArgLysMet	*C. perfringens*	8	59.67	1.218	1.365	1.713	0.403	2.115
High ArgLysMet	LPS	8	60.39	1.228	1.386	1.786	0.422	2.208
SEM			0.112	0.01	0.011	0.018	0.011	0.026
P-value								
Diet			0.956	0.002	0.024	0.197	0.065	0.081
Challenge			0.202	0.52	0.387	0.493	0.829	0.54
Diet × Challenge			0.293	0.341	0.829	0.878	0.661	0.772

^1^Low ArgLysMet, diets with low arginine, lysine and methionine levels; High ArgLysMet, diets with high arginine, lysine and methionine levels.

^2^At 25, 26, and 27 days of age, birds were challenged either with *C. perfringens* type A strain 56 (*C. perfringens*) or lipopolysaccharide from *Escherichia coli* (LPS), or served as a placebo group with no challenge (No). ^a,b^Means within a row with different superscripts differ significantly (*P* < 0.05).

**Table 2:** **Body weight gain (BWG) and feed conversion ratio (FCR) of turkeys calculated for pre-, during-, and post-challenge periods**

Parameter		N	BWG days 0–25, kg	BWG days 25–28, kg	BWG days 0–28, kg	FCR days 0–25, kg/kg	FCR days 25–28, kg/kg	FCR days 25–28, kg/kg
Diet^1^								
Low ArgLysMet		24	1.090^b^	0.205	1.639^b^	1.573	2.004	1.577
High ArgLysMet		24	1.150^a^	0.207	1.732^a^	1.53	2.242	1.567
Challenge^2^								
No		16	1.106	0.23	1.711	1.58	2	1.578
*C. perfringens*		16	1.118	0.213	1.662	1.527	2.039	1.518
LPS		16	1.136	0.176	1.684	1.548	2.33	1.62
Treatments								
Low ArgLysMet	No	8	1.094	0.213	1.648	1.571	1.988	1.563
Low ArgLysMet	*C. perfringens*	8	1.078	0.22	1.634	1.575	1.864	1.559
Low ArgLysMet	LPS	8	1.099	0.182	1.636	1.574	2.16	1.608
High ArgLysMet	No	8	1.118	0.248	1.774	1.59	2.012	1.593
High ArgLysMet	*C. perfringens*	8	1.159	0.206	1.691	1.479	2.214	1.477
High ArgLysMet	LPS	8	1.173	0.169	1.731	1.522	2.5	1.631
SEM			0.01	0.011	0.015	0.02	0.095	0.023
*P*-value								
Diet			0.003	0.91	0.002	0.28	0.217	0.838
Challenge			0.442	0.11	0.382	0.542	0.311	0.21
Diet × Challenge			0.416	0.562	0.612	0.496	0.731	0.542

^1^ Low ArgLysMet, diets with low arginine, lysine and methionine levels; High ArgLysMet, diets with high arginine, lysine and methionine levels.

^2^ At 25, 26, and 27 days of age, birds were challenged either with *C. perfringens* type A strain 56 (*C. perfringens*) or lipopolysaccharide from *Escherichia coli* (LPS), or served as a placebo group with no challenge (No). ^a,b^Means within a row with different superscripts differ significantly (*P* < 0.05).

### Gut integrity response to dietary treatments

#### Gut permeability

**Figure 1:**
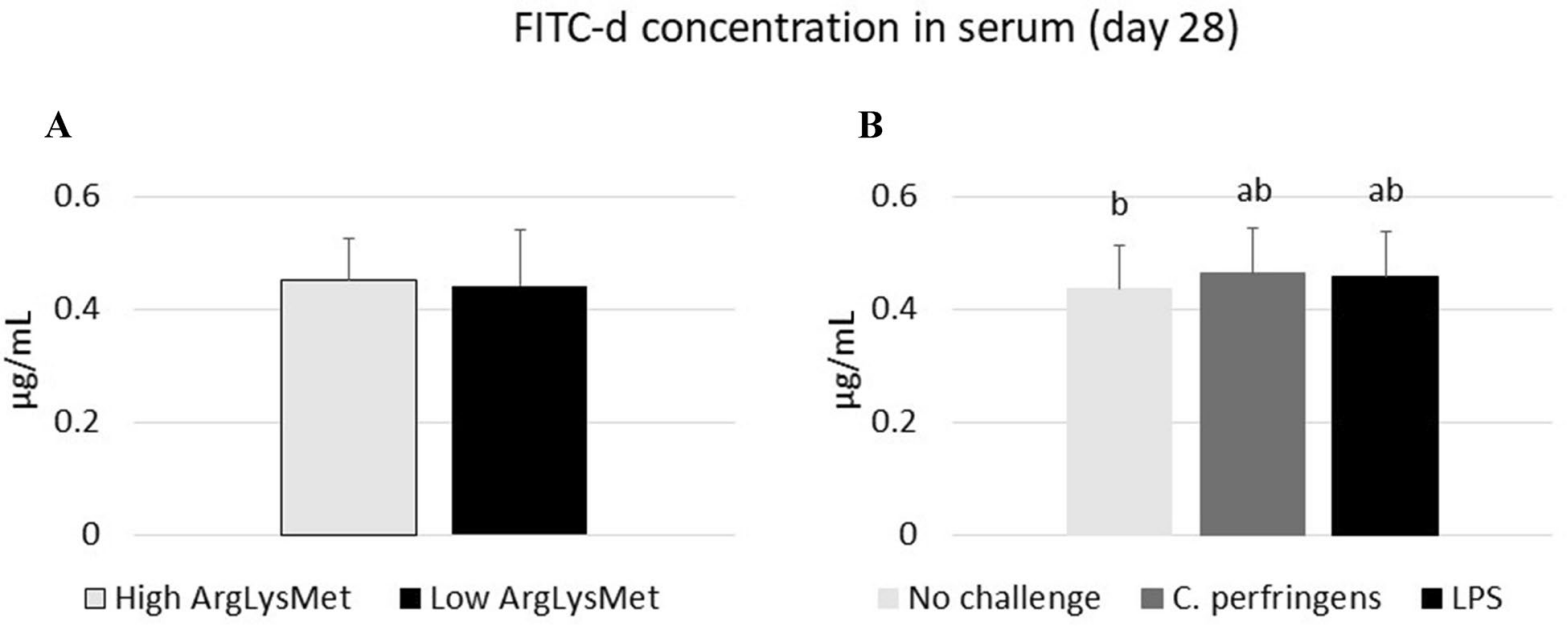
**Serum FITC-d concentrations in turkeys fed high or low dietary arginine, lysine and methionine levels (A) and as a result of either *C. perfringens* infection, *E. coli* LPS challenge or no challenge (B).** ^a,b^Means within a row with different superscripts differ significantly (*P* < 0.05).

The results of a gut permeability test are presented in Figure 1. Neither high nor low ArgLysMet diets significantly affected serum FITC-d concentrations in turkeys. Serum FITC-d concentrations were significantly higher in birds infected with *C. perfringens* than in uninfected birds (*P* = 0.027), whereas the administration of LPS did not induce significant differences relative to the control group.

#### Immune and redox status

Selected immunological and redox parameters in the blood plasma and jejunum of turkeys are presented in Tables 3 and 4. An analysis of plasma samples (Table 3) revealed a significant increase in 8-hydroxydeoxyguanosine (8-OHdG) concentration in response to low ArgLysMet diets (*P* < 0.001). An analysis of the small intestinal wall (Table 4) demonstrated that neither low nor high ArgLysMet diets affected the analyzed immunological and redox parameters; only oxoguanine glycosylase (OGG1) concentration increased in response to high ArgLysMet diets due to a significant diet × challenge interaction (*P* = 0.001). An analysis of immunological and redox parameters in the intestinal wall of turkeys (Table 4) revealed that the levels of dietary AAs significantly affected the concentrations of IgA (*P* < 0.001), IgY (*P* < 0.001), IL-6 (*P* = 0.027), Casp-8 (*P* = 0.008), OGG-1 (*P* = 0.015) and 8-OHdG (*P* < 0.001). In all cases, their effect was associated with a significant diet × challenge interaction. Low ArgLysMet diets increased IgA concentration in response to LPS, whereas high ArgLysMet diets exerted the opposite effect (*P* < 0.001). The concentration of IgY slightly decreased in turkeys fed low ArgLysMet diets, and considerably decreased in those fed high ArgLysMet diets (*P* = 0.029). The changes in IL-6 concentration were similar to those in IgA concentration (*P* = 0.034). The opposite relationships, also associated with the effect of LPS, were noted in the concentrations of Casp-8 (*P* = 0.022) and OGG-1 (*P* < 0.001). An analysis of the effect of infection, which was not associated with the diet × challenge interaction, revealed that LPS caused a significant increase in the concentrations of ceruloplasmin (Cp) (*P* = 0.046) and Casp-3 in the intestinal wall of turkeys (*P* = 0.049) (Table 4). An analysis of blood plasma samples (Table 3) revealed that the effect of infection, which was not associated with the diet × challenge interaction, caused an increase in the concentrations of endonuclease-1 (APEX-1) in birds infected with *C. perfringens* (*P* = 0.043) and 8-OHdG in birds challenged with *C. perfringens* or LPS, relative to unchallenged birds (*P* < 0.001); IgY concentration was lowest in turkeys administered LPS (*P* < 0.001); Cp concentration was higher in birds not infected with *C. perfringens* than in infected birds (*P* = 0.005). A significant diet × challenge interaction was noted for the plasma concentrations of IgA and IgM (*P* = 0.025 and *P* < 0.001, respectively), which decreased in LPS-challenged turkeys, compared with *C. perfringens*–challenged birds.

**Table 3:** **Selected immunological and redox parameters in the blood plasma of turkeys at 28 days of age**

Parameter		N	Ig A [ng/mL]	Ig M [ng/mL]	Ig Y [ng/mL]	IL-6 [pg/mL]	IL-2 [pg/mL]	Cp [ng/mL]	Casp3 [ng/mL]	Casp8 [ng/mL]	OGG1 [ng/mL]	APEX1 [ng/mL]	8-OHdG [ng/mL]
Diet^1^													
Low ArgLysMet		24	2.457^a^	2.759	2356.88	4.4	17.96	29.17	0.989	1.653	6.177	202.4	27.43^a^
High ArgLysMet		24	2.216^b^	2.669	2343.92	5.158	16.88	29.85	1.067	1.691	5.758	185.8	22.99^b^
Challenge													
No		16	2.399^a^	2.741^a^	2407.58^a^	5.244	17.29	23.41^b^	1.022	1.469	5.811	192.5^ab^	34.40^a^
*C. perfringens*		16	2.457^a^	2.882^a^	2406.39^a^	5.098	19.96	36.43^a^	1.039	1.874	5.997	220.8^a^	21.64^b^
LPS		16	2.153^b^	2.520^b^	2237.23^b^	3.994	15	28.69^ab^	1.023	1.672	6.095	169.1^b^	19.58^b^
Treatments^2^													
Low ArgLysMet	No	8	2.403^a^	2.891	2382.22	4.898	15.90^ab^	23.94	1.012	1.32	6.138	217.6	37.49
Low ArgLysMet	*C. perfringens*	8	2.595^a^	2.722	2394.5	4.723	13.49^ab^	33.69	0.936	1.846	6.492	226.3	25.54
Low ArgLysMet	LPS	8	2.373^a^	2.664	2293.92	3.58	24.48^a^	29.86	1.02	1.792	5.903	163.4	19.24
High ArgLysMet	No	8	2.395^a^	2.591	2432.95	5.591	18.69^ab^	22.88	1.032	1.619	5.483	167.3	31.32
High ArgLysMet	*C. perfringens*	8	2.319^a^	3.042	2418.28	5.474	26.44^a^	39.16	1.143	1.903	5.503	215.3	17.73
High ArgLysMet	LPS	8	1.933^b^	2.375	2180.53	4.408	5.51^b^	27.51	1.027	1.551	6.288	174.7	19.93
SEM			0.041	0.041	21.686	0.252	1.688	1.665	0.029	0.078	0.195	8.572	1.469
*P*-value													
Diet			0.001	0.115	0.728	0.13	0.707	0.824	0.181	0.803	0.294	0.31	0.049
Challenge			0.001	0.001	0.001	0.087	0.371	0.005	0.964	0.111	0.837	0.043	0.001
Diet × Challenge			0.025	0.001	0.164	0.994	0.001	0.541	0.295	0.365	0.343	0.304	0.253

^1^Low ArgLysMet, diets with low arginine, lysine and methionine levels; High ArgLysMet, diets with high arginine, lysine and methionine levels.

^2^At 25, 26, and 27 days of age, birds were challenged either with *C. perfringens* type A strain 56 (*C. perfringens*) or lipopolysaccharide from *Escherichia coli* (LPS), or served as a placebo group with no challenge (No). ^a,b^Means within a row with different superscripts differ significantly (*P* < 0.05).

**Table 4:** **Selected immunological and redox parameters in the jejunum of turkeys at 28 days of age**

Parameter		N	Amyloid A [ug/g]	Ig A [ng/g]	Ig M [ng/g]	Ig Y [ng/g]	IL-6 [pg/g]	IL-2 [pg/g]	Cp [ng/g]	Casp3 [ng/g]	Casp8 [ng/g]	OGG1 [ng/g]	APEX1 [ng/gL]	8-OHdG [ng/mL]
Diet^1^														
Low ArgLysMet		24	26.87	44.61	31.45	326.7	9.72	11.65	598.7	15.41	30.44	63.92^b^	3.243	24.44
High ArgLysMet		24	22.59	45.3	32.16	330.5	10.38	11.12	624.9	14.15	31.18	74.12^a^	3.553	22.92
Challenge^2^														
No		16	23.34	41.78^b^	32.63	335.0^a^	8.71^b^	10.94	562.2^b^	14.37	32.20^a^	72.73^a^	3.287	19.18^c^
*C. perfringens*		16	25.76	56.84^a^	33.59	385.2^a^	11.94^a^	12.30	624.0^ab^	14.03	27.63^b^	63.05^b^	3.11	24.37^b^
LPS		16	25.09	36.23^b^	29.19	265.6^b^	9.49^ab^	10.92	649.2^a^	15.94	32.60^a^	71.28^ab^	3.798	27.47^a^
Treatments														
Low ArgLysMet	No	8	19.44	34.68	30.60^ab^	318.7	7.08^b^	8.92	521.7	15.47	34.28	73.36	3.506	21.64
Low ArgLysMet	*C. perfringens*	8	23.25	52.31	29.82^ab^	360.3	11.10^ab^	10.77	617.4	14.93	24.88	63.96	2.894	23.82
Low ArgLysMet	LPS	8	37.93	46.83	33.91^ab^	301.2	10.98^ab^	15.27	656.9	15.83	32.15	54.44	3.33	27.84
High ArgLysMet	No	8	27.24	48.89	34.65^a^	351.4	10.35^ab^	12.96	602.7	13.27	30.12	72.09	3.067	16.73
High ArgLysMet	*C. perfringens*	8	28.26	61.38	37.35^a^	410	12.78^a^	13.83	630.5	13.13	30.37	62.14	3.327	24.92
High ArgLysMet	LPS	8	12.25	25.63	24.48^b^	230	7.99^ab^	6.57	641.4	16.05	33.04	88.11	4.266	27.1
SEM														
*P*-value			1.585	2.41	1.08	11.263	0.658	0.728	14.85	0.352	0.78	2.039	0.211	0.675
Diet			0.068	0.848	0.709	0.848	0.507	0.589	0.361	0.06	0.588	0.001	0.247	0.08
Challenge			0.001	0.001	0.151	0.001	0.027	0.423	0.046	0.049	0.008	0.015	0.100	0.001
Diet × Challenge			0.001	0.001	0.002	0.029	0.034	0.001	0.370	0.276	0.022	0.001	0.111	0.001

^1^Low ArgLysMet, diets with low arginine, lysine and methionine levels; High ArgLysMet, diets with high arginine, lysine and methionine levels.

^2^At 25, 26, and 27 days of age, birds were challenged either with *C. perfringens* type A strain 56 (*C. perfringens*) or lipopolysaccharide from *Escherichia coli* (LPS), or served as a placebo group with no challenge (No). ^a,b^Means within a row with different superscripts differ significantly (*P* < 0.05).

**Figure 2:**
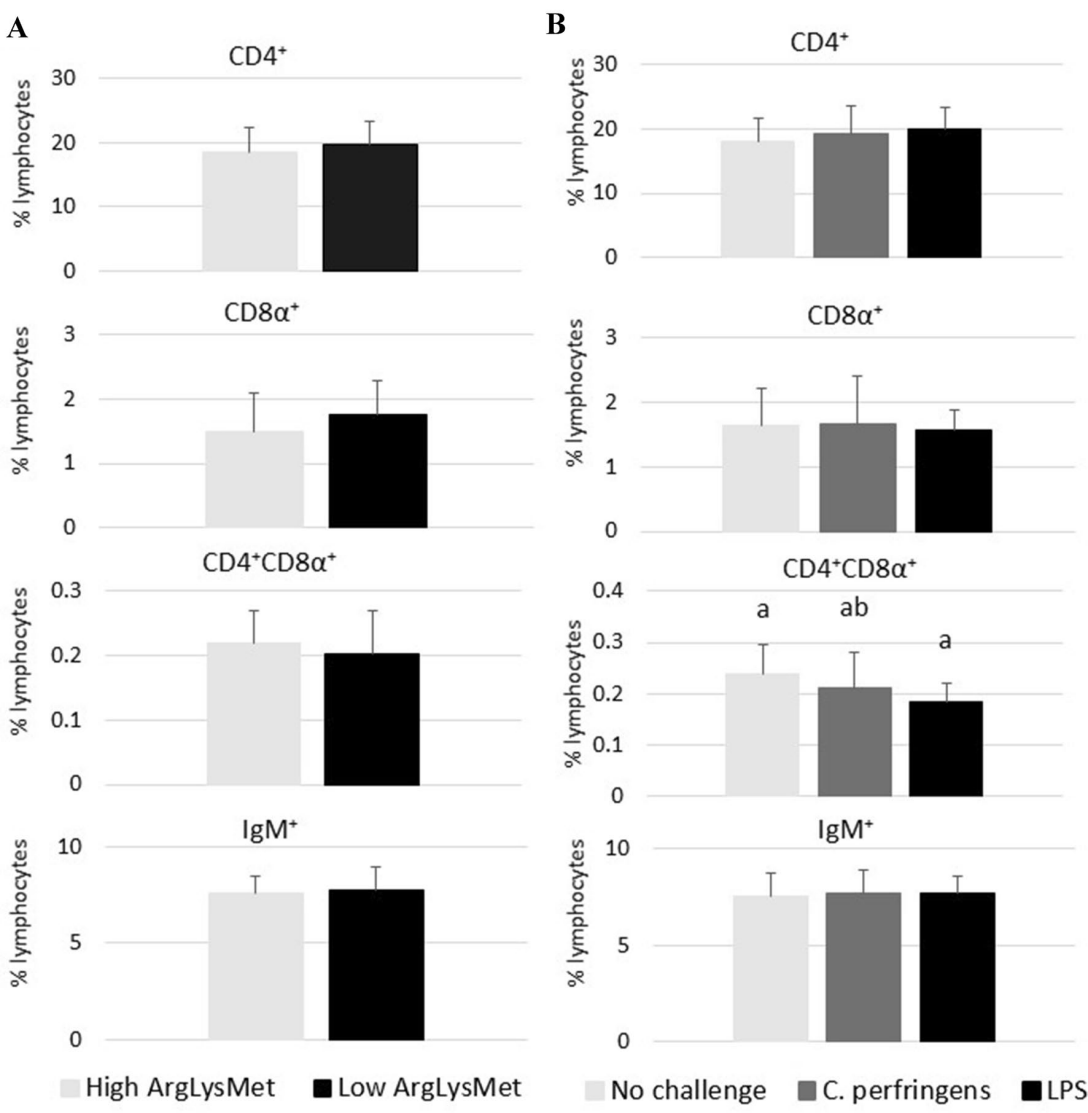
**Percentages of peripheral blood T (CD4**^**+**^**, CD4**^**+**^**CD8α**^**+**^**, CD8α**^**+**^**) and B (IgM**^**+**^**) cell subpopulations in turkeys fed high or low dietary arginine, lysine and methionine levels (left side of the figure) and as a result of either *C. perfringens* infection, *E. coli* LPS challenge or no challenge (right side of the figure).** ^a,b^Means within a row with different superscripts differ significantly (*P* < 0.05). A significant interaction (diet × challenge) was noted (*P* = 0.047) for CD4+ CD8α+.

**Figure 3:**
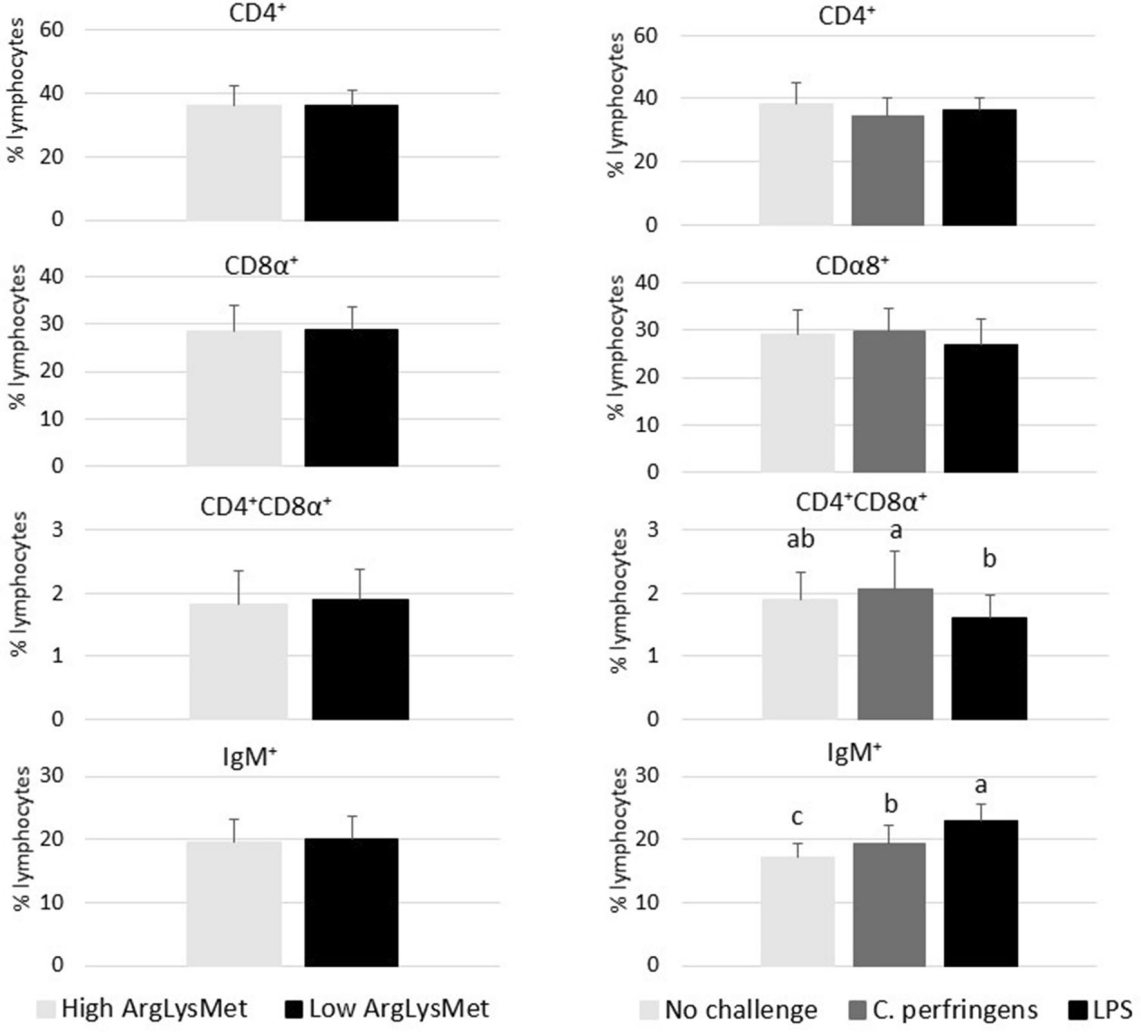
**Percentages of splenic T (CD4**^**+**^**, CD4**^**+**^**CD8α**^**+**^**, CD8α**^**+**^**) and B (IgM**^**+**^**) cell subpopulations in turkeys fed high or low dietary arginine, lysine and methionine levels (left side of the figure) and as a result of either *C. perfringens* infection, *E. coli* LPS challenge or no challenge (right side of the figure).** ^a,b,c^Means within a row with different superscripts differ significantly (*P* < 0.05).

The percentages of T (CD4^+^, CD8α^+^, CD4^+^CD8α^+^) and B (IgM^+^) cell subpopulations in the blood and spleen of turkeys are presented in Figures 2 and 3, respectively. The dietary inclusion levels of Arg, Lys and Met had no significant effect on the percentages of the analyzed cell subpopulations (*P* > 0.05). In turn, the percentage of CD4^+^CD8α^+^ double positive T cell subpopulations in the spleen of turkeys was significantly affected by infection, and it was higher in birds challenged with *C. perfringens* than in those administered LPS (*P* = 0.032). The percentage of IgM^+^ B subpopulations was significantly higher in turkeys receiving LPS than in those challenged with *C. perfringens* (*P* < 0.001). Infection induced significant changes in the percentage of CD4^+^CD8α^+^ T cell subpopulations in the blood of turkeys (*P* = 0.021). A significant diet × challenge interaction was observed (*P* = 0.047): infection with *C. perfringens* decreased their percentage in turkeys fed low ArgLysMet diets, and increased their percentage in birds fed high ArgLysMet diets.

### Response of the transcript levels of selected genes encoding gut integrity, nutrient transporters and digestive enzymes to dietary treatments

The mRNA expression patterns of selected genes encoding gut integrity regulators and nutrient transporters in the jejunum are presented in Table 5. In general, dietary AA levels had a significant effect on the mRNA expression of selected transporter genes. The expression level of the PEPT 1 gene decreased (*P* = 0.034), and the expression level of the PEPT 2 gene increased (*P* = 0.025) in response to high ArgLysMet diets. High ArgLysMet diets also decreased the expression level of the SI gene (*P* = 0.007), but they had no influence on the expression levels of genes encoding tight junction proteins (TJPs). The infectious agents exerted a greater effect on the expression levels of both transporter genes and genes encoding TJPs (Figure 3). Infections with both *C. perfringens* and LPS decreased the expression levels of GLUT 2 (*P* < 0.001), PEPT 1 (*P* < 0.001), SI (*P* < 0.001) and EAAT 3 (*P* < 0.001) genes. The expression levels of ASCT 1 (*P* < 0.001), OCCL (*P* < 0.001) and CAT 1 (*P* < 0.001) genes also decreased in response to the stressors, but significant diet × challenge interactions were observed in this case. The expression level of the ASCT 1 gene was not affected by low ArgLysMet diets, whereas in turkeys fed high ArgLysMet diets, infection with *C. perfringens* or LPS decreased the expression level of this gene, relative to unchallenged birds (*P* = 0.045). The expression level of the OCCL gene decreased in response to *C. perfringens* infection, and the noted decrease was greater in turkeys fed high ArgLysMet diets than in birds receiving low ArgLysMet diets (*P* = 0.005). The expression pattern of the CAT 1 gene was identical in turkeys challenged with LPS (*P* = 0.007).

**Table 5:** **mRNA expression levels of selected genes encoding gut integrity regulators, nutrient transporters and digestive enzymes in turkeys at 28 days of age**

Parameter		N	GLUT 1	GLUT 2	PEPT 1	PEPT 2	ASCT 1	ZO 1	OCCL	BoAT	SI	EAAT 3	CCK 1	CAT 1	CCK
Diet^1^															
Low ArgLysMet		24	0.812	0.236	0.196^a^	0.789^b^	0.928	0.565	0.611	0.874	0.788^a^	0.301	6.551	1.000	2.368
High ArgLysMet		24	0.803	0.226	0.147^b^	1.107^a^	0.928	0.56	0.698	0.66	0.563^b^	0.33	6.499	1.076	2.075
Challenge^2^															
No		16	0.865	0.390^a^	0.271^a^	1.126	1.187^a^	0.648	0.919^a^	1.003	0.946^a^	0.502^a^	6.144	1.433^a^	1.732
*C. perfringens*		16	0.736	0.155^b^	0.135^b^	0.757	0.728^b^	0.494	0.424^c^	0.686	0.573^b^	0.183^b^	5.654	0.988^b^	2.569
LPS		16	0.821	0.148^b^	0.109^b^	0.962	0.868^b^	0.545	0.620b	0.612	0.508^b^	0.262^b^	7.777	0.692^c^	2.363
Treatments															
Low ArgLysMet	No	8	0.699	0.380	0.313	0.939	1.035^ab^	0.606	0.747	1.202	1.162	0.495	4.878	1.200	1.936
Low ArgLysMet	*C. perfringens*	8	0.769	0.188	0.144	0.826	0.768^b^	0.506	0.473	0.794	0.703	0.225	5.914	0.946	2.986
Low ArgLysMet	LPS	8	0.968	0.138	0.132	0.601	0.980^ab^	0.583	0.613	0.627	0.499	0.185	8.86	0.854	2.181
High ArgLysMet	No	8	1.03	0.399	0.229	1.312	1.340^a^	0.691	1.091	0.804	0.73	0.51	7.41	1.666	1.529
High ArgLysMet	*C. perfringens*	8	0.703	0.121	0.126	0.687	0.688^b^	0.481	0.375	0.578	0.443	0.142	5.393	1.031	2.151
High ArgLysMet	LPS	8	0.674	0.159	0.085	1.323	0.756^b^	0.508	0.627	0.597	0.517	0.339	6.694	0.531	2.546
SEM			0.043	0.020	0.015	0.077	0.051	0.026	0.042	0.078	0.051	0.03	0.459	0.068	0.152
*P*-value															
Diet			0.906	0.668	0.034	0.025	0.997	0.928	0.117	0.165	0.007	0.526	0.953	0.435	0.317
Challenge			0.421	0.001	0.001	0.101	0.001	0.053	0.001	0.095	0.001	0.001	0.131	0.001	0.058
Diet × Challenge			0.010	0.171	0.501	0.045	0.037	0.433	0.005	0.618	0.074	0.110	0.099	0.007	0.240

^1^Low ArgLysMet, diets with low arginine, lysine and methionine levels; High ArgLysMet, diets with high arginine, lysine and methionine levels.

^2^At 25, 26, and 27 days of age, birds were challenged either with *C. perfringens* type A strain 56 (*C. perfringens*) or lipopolysaccharide from *Escherichia coli* (LPS), or served as a placebo group with no challenge (No). ^a,b,c^Means within a row with different superscripts differ significantly (*P* < 0.05). GLUT 1: Glucose transporter-1, GLUT 2: Glucose transporter-2, PEPT 1: Peptide transporter-1, PEPT 2: Peptide transporter-2, ASCT 1: Alanine, serine, cysteine, and threonine transporter, ZO 1: Zonula occludens 1, OCCL: Occludin, BoAT: Solute carrier family 6, member 19, SI: Sucrase isomaltase, EAAT 3: Excitatory amino acid transporter 3, CCK 1: Cholecystokinin type 1 receptor, CAT 1: Cationic amino acid transporter-1, CCK: Cholecystokinin.

## Discussion

The present results regarding birds performance response are partially consistent with the findings of Oso et al. [26], who reported that Arg-supplemented diets increased the BW of 16-week-old turkeys. In another study of turkeys [27], increased dietary Lys content, relative to NRC recommendations [12], significantly affected the FCR during the first 4 weeks of rearing. The present results partially confirm the fact that the dietary Arg:Lys ratio is of key importance because these AAs have similar structure and perform similar functions in the body [28]; therefore, diets with varying proportions of Arg and Lys exert the greatest effects on bird performance [13]. Our previous study [29] revealed that increased dietary levels of Arg and Lys had no significant influence on the growth performance of turkeys. In the current experiment, high ArgLysMet diets improved bird performance, which may suggest that the optimal ratios of all three AAs are most effective in maintaining high productivity. The present study also demonstrated that challenge with *C. perfringens* did not compromise the growth performance of turkeys. This indicates that subclinical NE after experimental infection with *C. perfringens* does not affect the BW or BWG of birds [30]. Infected turkeys that do not display disease symptoms may pose a potential health risk as vectors that carry and transmit pathogens to the food chain, because such birds are not treated or culled. In this experiment, an anatomopathological analysis of the intestines during a post-mortem examination (data not shown) revealed that most of the observed changes (hyperemia, yellow coating on the mucosa, general appearance of the intestinal wall and digesta) were not characteristic of NE associated with *C. perfringens* infection. The administration of *E. coli* LPS did not cause intestinal mucosa damage visible to the naked eye, either.

Gut permeability has been considered as indicator of gut integrity [31]. Fluorescein-5-isothiocyanate dextran, a non-digestible polysaccharide with an average molecular weight of 4 kDa, is widely used as a biomarker to measure intestinal paracellular permeability in vivo. When administered *per os*, it does not cross the intestinal epithelial barrier in high quantities unless the barrier is compromised [31]. Intestinal barrier integrity is essential for nutrient absorption and the maintenance of normal bodily function. Intestinal barrier disfunction increases gut permeability, leading to pathological conditions in the GIT. In this respect, a key role is played by limiting AAs such as Arg, Lys and Met, which serve as substrates for protein biosynthesis and reinforce intestinal barrier function [32]. Both excess and deficiency of dietary AAs may lead to intestinal barrier dysfunction and the development of various diseases in birds raised under optimal or stress conditions [10]. In the present experiment, high ArgLysMet diets did not contribute to increased gut permeability. Oso et al. [26] also found that Arg-supplemented diets had a beneficial influence on selected parameters of intestinal morphology in turkeys (increased intestinal villus height and crypt depth). Foye et al. [33] noted improved nutrient absorption in turkey poults fed Arg *in ovo*. In contrast, Barekatain et al. [34] demonstrated that broiler chickens fed diets with reduced protein (and limiting AA) concentrations had higher serum FITC-d concentrations, pointing to increased intestinal permeability, compared with birds fed high-protein diets. The differences between these findings and our results could be due to the fact that growing chickens and turkeys differ considerably in their AA requirements (Aviagen [35] vs. Hendrix Genetics [16]). Our previous study revealed that increased dietary inclusion levels of Lys were more effective than Arg in modulating the functional status of the gut in turkeys by decreasing the pH of cecal digesta, enhancing the synthesis of butyric acid and decreasing the concentrations of putrefactive short-chain fatty acids and ammonia in the cecum [29]. However, the simultaneous effects of high or low dietary rates of all three AAs (Arg, Lys and Met) on gut permeability in turkeys have not been investigated to date. Interestingly, the present findings point to differences between the analyzed stressors since infection with *C. perfringens* resulted in greater intestinal permeability than the administration of *E. coli* LPS. The noted differences were due to the different modes of action of live *C. perfringens* bacteria and LPS (endotoxin) isolated from *E. coli* in the host’s body [36]. In broiler chickens, *E. coli* LPS stimulated the production of proinflammatory cytokines (IL-1, IL-6) and TNF-α by macrophages [37], whereas *C. perfringens* reduced matrix metalloproteinase activity in the jejunal mucosa [38].

Another factor closely related to gut barrier function is immune and redox status. The present results regarding blood immune and redox status of birds indicate that under optimal conditions (in the absence of stressors), increased dietary inclusion levels of Arg, Lys and Met did not over-stimulate the immune system and did not disrupt the redox balance in the GIT. It should be noted that in contrast to other immunostimulatory substances, AAs do not increase the demand for energy and nutrients (in particular protein) to maintain the immune responses of birds that had been triggered unnecessarily [39]. Therefore, they do not contribute to chronic stimulation that reduces the efficiency of the immune system. Analysis of the immune and redox status of the small intestinal wall of birds do not corroborate the results of our previous study [40] where turkeys were fed diets with different Arg:Lys ratios relative to Met. In the cited study, no significant interactions were found between different dietary proportions of Arg and Lys vs. the immune response and the antioxidant status in the intestinal wall and blood of turkeys. In another experiment [17], where diets differed also in Met content and turkeys were infected with *C. perfringens*, diet × challenge interactions were observed more frequently, similarly to the present study. In this study, turkeys were also exposed to another pathological factor, i.e. LPS isolated from *E. coli* cell walls. It was found that the immune and oxidative responses of young turkeys varied depending on the stressor (*C. perfringens* vs. LPS), and further research is needed to explore those relationships. The host’s response to LPS was more spontaneous, which could be due to the fact that this stressor exerted both toxic and immunomodulatory (adjuvant) effects on the gut-associated immune system in birds [36, 41].

Double positive T cells (CD4+CD8+) play a central role in peripheral tissues as strong suppressors of immunity and as cells with high cytotoxic potential [42, 43]. Similarly to redox status markers, no significant differences in immune system function were observed in turkeys fed diets with different inclusion levels of Arg, Lys and Met, which indicates that the applied dietary treatments had no negative effect on the health status of birds. In a study by De Jonge et al. [44], Arg deficiency compromised B cell proliferation in the spleen of transgenic mice. Li et al. [45] found that Arg played a key role in the proliferation of T and B cells in poultry. According to Calder [46], Arg is required for the synthesis of immune system proteins in turkeys, similarly to Met [47, 48], which is why in this experiment neither high nor low ArgLysMet diets had a negative effect on the percentages of the analyzed T and B cell subpopulations in the spleen of turkeys. Other studies demonstrated that increased dietary Met content (by 0.60% relative to the control group where the recommended level of Met was applied) contributed to an increase in Met concentration in peripheral blood and in the percentages of CD4^+^CD8α^+^ T cell subpopulation in the thymus and the bursa of Fabricius in experimental birds [24, 49]. In the present study, differences were noted in the percentages of CD4^+^CD8α^+^ T cell and IgM^+^ B cell subpopulations in the spleen of turkeys in response to the challenge (*C. perfringens* vs. LPS); infection with *C. perfringens* led to a greater increase in the percentage of CD4^+^CD8α^+^ T cell subpopulations than the administration of LPS, whereas the opposite was observed in IgM^+^ B cells. The above differences most probably resulted from the fact that *C. perfringens* and LPS exerted different effects on the immune system of turkeys. It was found that proinflammatory cytokines, including IL-6, participate in the initiation of T and B cell proliferation in response to stressors [50].

Nutrient transporters are proteins responsible for the transportation of AAs into and out of cells. Aminopeptidase cleaves AAs from the N-terminus of polypeptides, making them available for transportation, whereas PEPT 1 is a di- and tripeptide transporter. In the intestine, these proteins are located on the brush border and the basolateral membranes of enterocytes, and they are involved in the uptake of AAs by enterocytes and their release into circulation or distribution among other cells [51]. Individual AAs are absorbed by various Na+ dependent and independent transport systems [52]. However, most AAs from proteins transported to the intestines are absorbed as dipeptides and tripeptides rather than individually. Dipeptides and tripeptides are absorbed more rapidly and efficiently via the PEPT 1 transporter than individual AAs [53]. The expression of the PEPT 1 gene was confirmed in poultry [54, 55], but further research is needed to elucidate the role of the PEPT 1 transporter in intestinal AA capture in birds. In the current experiment, the expression level of the PEPT 1 gene decreased in jejunal tissue in response to high ArgLysMet diets. Most probably, this resulted from the fact that larger amounts of available AAs (Arg Lys Met) were transported to the small intestine, leading to slower release of free AAs, which reduced the need for increased expression of the PEPT 1 gene in the intestine. Similar relationships were observed in broiler chickens by Gilbert et al. [56]. Another important transporter is PEPT 2, a high-affinity/low-capacity transporter that translocates dipeptides and tripeptides [57]. In the current study, high ArgLysMet diets increased the expression level of the PEPT 2 gene in small intestinal tissue. In broiler chickens, the expression level of the PEPT 2 gene increased significantly in the bursa of Fabricius at 14 days of age [58]. PEPT 2 can transport peptides to developing tissues, and peptide-based vaccines can boost the immune system [59]. It has also been found that PEPT 2 is expressed in the brain, kidneys, GIT, liver and lungs of birds during late embryogenesis, which suggests that it can act as an embryonic peptide transporter [60]. In the present experiment, high ArgLysMet diets decreased the expression of the gene encoding enzyme SI that is responsible for releasing glucose and fructose from nutrients. The correlation between high and low dietary levels of Arg, Lys and Met and the formation of functional enzyme SI has not been fully elucidated. It can be speculated that when a larger pool of AAs reaches the small intestine, changes in the expression levels of genes encoding the enzyme involved in glucose and fructose release are not needed due to the greater availability of AAs, because their transportation requires the expenditure of energy [61]. In the present study, changes in the expression levels of the analyzed genes were most pronounced in challenged turkeys. The expression levels of most genes encoding nutrient transporters, enzymes and TJPs were modified, and they decreased in the small intestinal tissue in all cases. This implies that even mild infection induces significant changes at the molecular level, although the growth performance of birds is not compromised, which corroborates the findings of Olkowski et al. [30]. Proteins of the occludin group (OCCL) are particularly important for maintaining intestinal barrier integrity because they are integral membrane proteins in tight junctions [62]. They form a tight barrier around cells and act as a physical barrier to the free flow of nutrients through intercellular spaces. Our previous study [17] revealed that *C. perfringens* bacteria can compromise the intestinal barrier by disintegrating TJPs in turkeys, which leads to malabsorption. The current study demonstrated that LPS can exert similar effects. In an experiment involving broiler chickens, the applied stressor (infection with *Campylobacter jejuni*) compromised nutrient transporter expression including GLUT 2, EAAT 3 and CST 1 in the small and large intestines [63]. Teng et al. [64] reported that the expression levels of genes encoding biomarkers associated with intestinal integrity and nutrient transporters decreased in broiler chickens infected with *Eimeria Maxima*.

The results of the present study demonstrated that increased dietary levels of Arg, Lys and Met had a beneficial effect on turkey performance and immunological parameters, and it improved selected indicators responsible for maintaining gut integrity in different challenge conditions. Under optimal conditions (with no challenge), high ArgLysMet diets maintained bird performance and they improved selected performance parameters in challenged birds. The immune system of turkeys was not excessively stimulated by high ArgLysMet diets, which did not disrupt the redox balance and had no negative effect on gut integrity. High ArgLysMet diets increased the expression levels of selected genes encoding nutrient transporters and TJPs. However, the influence exerted by different dietary inclusion levels of Arg, Lys and Met on gut integrity was largely interactive and determined by the stressor (*C. perfringens* vs. LPS). Further studies are required to investigate the role of Arg, Lys and Met levels in the diet on the immune response, gut function and performance of turkeys in different challenge conditions.

## Supplementary Information

**Additional file 1. Experiment layout.** Low ArgLysMet, diets with low arginine, lysine and methionine levels; High ArgLysMet, diets with high arginine, lysine and methionine levels. At 25, 26, and 27, days of age, birds were challenged either with *C. perfringens* type A strain 56 (*C. perfringens*) or lipopolysaccharide from *Escherichia coli* (LPS), or served as a placebo group with no challenge.

**Additional file 2. Ingredient composition and nutrient content of basal diets (g/100 g, as-fed basis) fed to turkeys at 1–28 days of age.**

**Additional file 3. Gating strategy for extracellular staining for CD4**^**+**^**, CD8α**^**+**^**, CD4**^**+**^**CD8α**^**+**^** and IgM**^**+**^** cells in spleen sample of the examined turkeys.** Abbreviations; FSC-A: forward scatter area, FSC-H: forward scatter height SSC-A: side scatter area.

**Additional file 4. Genes and primers used in the study.**

## Data Availability

The datasets used and/or analyzed during the current study are available from the corresponding author on reasonable request.

## Abbreviations

8-OHdG: 8-Hydroxydeoxyguanosine
ALP: albumin
ALT: alanine aminotransferase
APE-1: endonuclease 1
Arg: arginine
BWG: body weight gain
CK: creatine kinase
GLP 2: glucagon-like peptide 2
GPx: glutathione peroxidase
LPS: *E. coli* Lipopolysaccharide
Lys: lysine
Met: methionine
NE: necrotic enteritis
NSP: non-starch polysaccharides
OCCL: occludin
SOD: superoxide dismutase
TC: total cholesterol
TG: triglycerides
TJPs: tight junction proteins
TP: total protein
UA: uric acid
ZO 1: Zonula occludens 1
