# Supplementary figures and images for: Increased arginine, lysine, and methionine levels can improve the performance, gut integrity and immune status of turkeys but the effect is interactive and depends on challenge conditions
Source: Vet Res. 2022 Jul 26;53:59. doi: 10.1186/s13567-022-01080-7 (PMC9327309; doi:10.1186/s13567-022-01080-7)

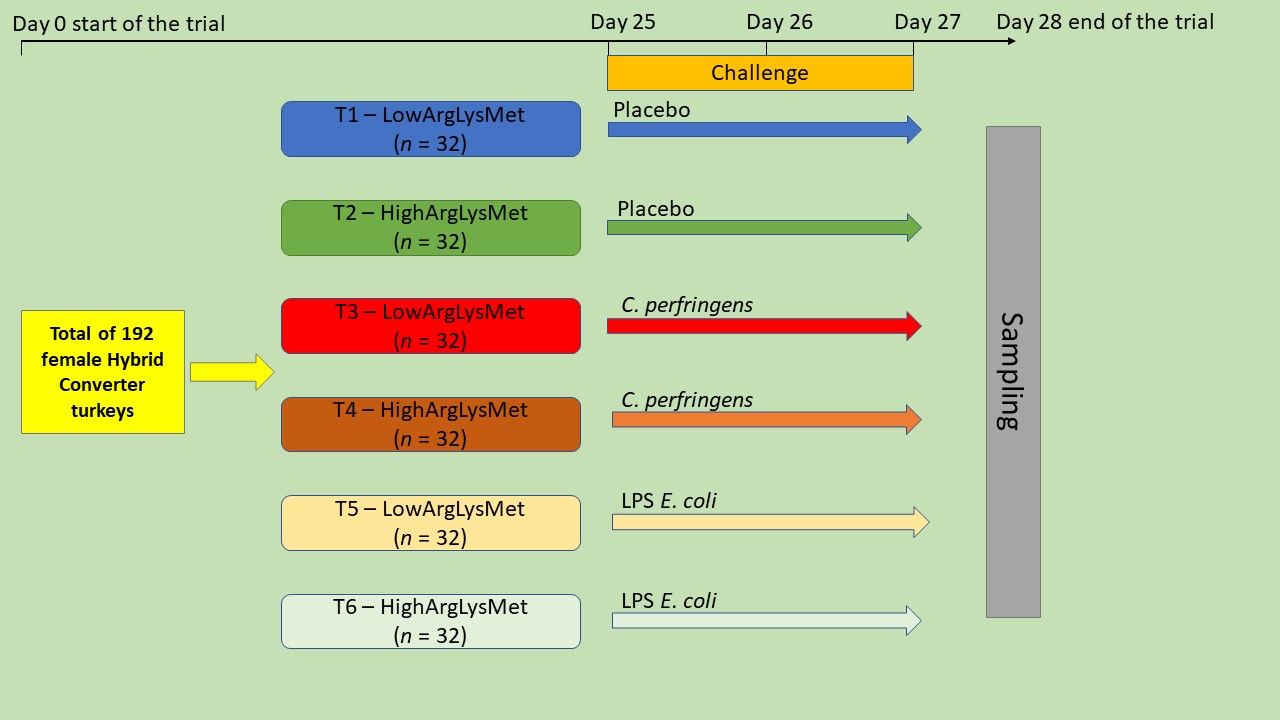

Supplement: Supplementary file 1 — Additional file 1. Experiment layout. Low ArgLysMet, diets with low arginine, lysine and methionine levels; High ArgLysMet, diets with high arginine, lysine and methionine levels. At 25, 26, and 27, days of age, birds were challenged either with C. perfringens type A strain 56 (C. perfringens) or lipopolysaccharide from Escherichia coli (LPS), or served as a placebo group with no challenge. [file 13567_2022_1080_MOESM1_ESM.jpg]

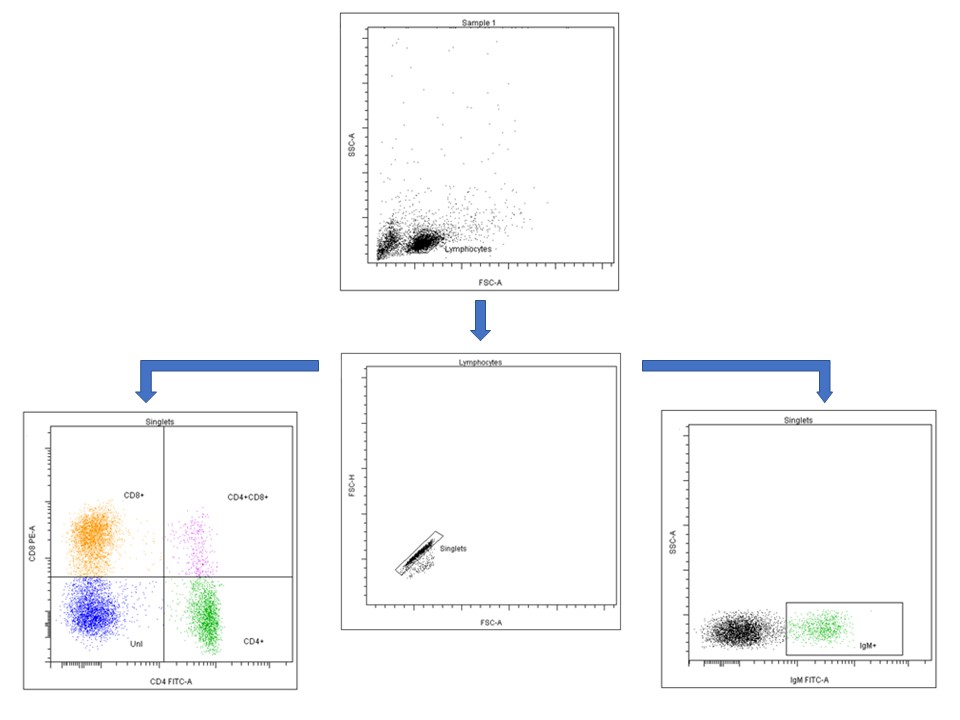

Supplement: Supplementary file 3 — Additional file 3. Gating strategy for extracellular staining for CD4+, CD8α+, CD4+CD8α+ and IgM+ cells in spleen sample of the examined turkeys. Abbreviations; FSC-A: forward scatter area, FSC-H: forward scatter height SSC-A: side scatter area. [file 13567_2022_1080_MOESM3_ESM.jpg]
