# Supplementary material for: Increased arginine, lysine, and methionine levels can improve the performance, gut integrity and immune status of turkeys but the effect is interactive and depends on challenge conditions
Source: Vet Res. 2022 Jul 26;53:59. doi: 10.1186/s13567-022-01080-7 (PMC9327309; doi:10.1186/s13567-022-01080-7)
Supplement: Supplementary file 2 — Additional file 2. Ingredient composition and nutrient content of basal diets (g/100 g, as-fed basis) fed to turkeys at 1–28 days of age. [file 13567_2022_1080_MOESM2_ESM.docx]

**Additional file 2. Ingredient composition and nutrient content of basal diets (****g/100 g, as-fed basis) fed to turkeys at 1–28 days of age**

| Parameter | Low ArgLysMet | High ArgLysMet |
| --- | --- | --- |
| Ingredients |  |  |
| Wheat | 56.736 | 59.069 |
| Soybean meal, 46% CP | 19.956 | 16.627 |
| Rapeseed meal | 6.0 | 6.0 |
| Potato protein | 5.45 | 5.45 |
| Soybean oil | 1.059 | 0.56 |
| Maize gluten meal | 6.0 | 6.0 |
| Sodium bicarbonate | 0.2 | 0.2 |
| Sodium chloride | 0.155 | 0.158 |
| Limestone | 1.920 | 1.938 |
| Monocalcium phosphate | 1.469 | 1.487 |
| L Lysine HCL | 0.495 | 0.855 |
| DL Methionine | - | 0.379 |
| Arginine | - | 0.667 |
| L-Threonine | 0.061 | 0.111 |
| Vitamin-mineral premix^1^ | 0.5 | 0.5 |
| Calculated nutrient content |  |  |
| Metabolizable energy, kcal/kg | 2880 | 2880 |
| Crude protein | 26.5 | 26.5 |
| Crude fat | 3.485 | 2.999 |
| Met + Cys | 0.922 | 1.264 |
| Threonine | 1.05 | 1.05 |
| Calcium | 1.25 | 1.25 |
| Available phosphorus | 0.65 | 0.65 |
| Arginine | 1.41 | 1.98 |
| Lysine | 1.60 | 1.80 |
| Methionine | 0.451 | 0.81 |
| Analyzed AA content |  |  |
| Arginine | 1.50 | 1.97 |
| Lysine | 1.74 | 1.97 |
| Methionine | 0.63 | 0.89 |

^1^Provided per kg of diet: mg: retinol 3.78, cholecalciferol 0.13, α-tocopheryl acetate 100, vit. K_3_ 5.8, thiamine 5.4, riboﬂavin 8.4, pyridoxine 6.4, cobalamin 0.032, biotin 0.32, pantothenic acid 28, nicotinic acid 84, folic acid 3.2, Fe 64, Mn 120, Zn 110, Cu 23, I 3.2, Se 0.30.
