# Supplementary material for: Increased arginine, lysine, and methionine levels can improve the performance, gut integrity and immune status of turkeys but the effect is interactive and depends on challenge conditions
Source: Vet Res. 2022 Jul 26;53:59. doi: 10.1186/s13567-022-01080-7 (PMC9327309; doi:10.1186/s13567-022-01080-7)
Supplement: Supplementary file 4 — Additional file 4. Genes and primers used in the study. [file 13567_2022_1080_MOESM4_ESM.docx]

**Additional file 4. Genes and primers used in the study**

| Gene name | Abbreviation/sequence | Sequence (5'-3') | Product length | Melting temperature (°C) | GenBank access no. |
| --- | --- | --- | --- | --- | --- |
| Phosphoglycerate Kinase 1 | PGK1 Reverse | GCCATCAGGTCCTTGACAAT | 167 | 63.9 | NM_204985 |
|  | PGK1 Forward | AAAGTTCAGGATAAGATCCAGCTG |  | 63.5 |  |
| Transferrin Receptor | TFRC Reverse | GTAGCACCCACAGCTCCGT | 113 | 65.4 | NM_205256 |
|  | TFRC Forward | GGAACTTGCCCGTGTGATC |  | 65.6 |  |
| Ribosomal Protein S7 | RPS7 Reverse | TTGGCTTGGGCAGAATCC | 156 | 66.2 | XM_419936 |
|  | RPS7 Forward | TAGGTGGTGGCAGGAAAGC |  | 65.2 |  |
| Cationic amino acid transporter-1 | CAT1 Reverse | GCCACACTGGAGGTTCCGAT | 152 | 68.6 | XM_003203401.4 |
|  | CAT1 Forward | CTGGCTGGACTCTGCTATGG |  | 64.9 |  |
| Alanine, serine, cysteine, and threonine transporter | ASCT1 Reverse | GTTGCCGTGACGAGAATGGT | 116 | 67.4 | XM_010706509.3 |
|  | ASCT1 Forward | ACTGTGAACATGGACGGAGC |  | 65.3 |  |
| Peptide transporter-1 | Pept1 Reverse | AGAACATACTCTGCCCACTGC | 138 | 63.2 | NM_001303166.1 |
|  | Pept1 Forward | TTCATACTCACAGGCACCATC |  | 62.6 |  |
| Peptide transporter-2 | Pept2 Reverse | CCAACCATAGACAGGACCACAT | 143 | 64.8 | XM_010713884.3 |
|  | Pept2 Forward | ATCATGGCAGACTCGTGGTT |  | 64.6 |  |
| Cholecystokinin | CCK Reverse | CCAGCCCATGTAGTCTCTGTC | 116 | 63.7 | XM_003207259.4 |
|  | CCK Forward | CCAAGTACCTGCAGCAAGCC |  | 67.0 |  |
| Cholecystokinin type 1 receptor | CCK1 Reverse | CACCAGGTAGCAGCAATCAC | 102 | 63.4 | XM_003205880.4 |
|  | CCK1 Forward | TGCCATCTCTTTGGAGCGG |  | 68.7 |  |
| Excitatory amino acid transporter 3 | EAAT3 Reverse | ATGCCAACTCCAATACCCAGC | 171 | 67.0 | XM_010725627.3 |
|  | EAAT3 Forward | GAGAAGGGCGGTTCGACTC |  | 66.2 |  |
| Glucose transporter-1 | GLUT1 Revese | GCTGCTCCACACCTGACTTC | 99 | 65.1 | XM_003212361.4 |
|  | GLUT 1 Forward | TCATCGCAATCGTGTTGCAG |  | 68.4 |  |
| Glucose transporter-2 | GLUT2 Reverse | CCCGTAGTGCGCTTCTATC | 104 | 62.4 | XM_010716927.3 |
|  | GLUT 2 Forward | CGTTGGTCCTCTCCGTCTTC |  | 66.1 |  |
| Solute carrier family 6, member 19 | BoAT Reverse | GCACGCCAGCGATGATTAC | 177 | 66.7 | XM_010708607.3 |
|  | BoAT Forward | GGAGAGTTATCAGTCCGCTCA |  | 63.3 |  |
| Sucrase isomaltase | SI Reverse | AGCCAGAATGTAACCGCCTC | 161 | 65.3 | XM_031555006.1 |
|  | SI Forward | TAATGACTCCGGGTGCCGT |  | 67.7 |  |
| Zonula occludens 1 | ZO 1 Reverse | CTGCTGAGAGGCTAATACAA | 114 | 57.3 | XM_019619275 |
|  | ZO 1 Forward | AGAGGCAACTGAACCATAG |  | 56.8 |  |
| Occludin | Occl Reverse | GTTCACACTCACCTCCTG | 127 | 56.3 | XM_019610822 |
|  | Occl Forward | GCAGATGTCCAGCAGTTA |  | 57.3 |  |

PGK 1: Phosphoglycerate kinase 1, TFRC: Transferrin receptor, RPS 7: Ribosomal Protein S7, GLUT 1: Glucose transporter-1, GLUT 2: Glucose transporter-2, PEPT 1: Peptide transporter-1, PEPT 2: Peptide transporter-2, ASCT 1: Alanine, serine, cysteine, and threonine transporter, ZO 1: Zonula occludens 1, OCCL: Occludin, BoAT: Solute carrier family 6, member 19, SI: Sucrase isomaltase, EAAT 3: Excitatory amino acid transporter 3, CCK 1: Cholecystokinin type 1 receptor, CAT 1: Cationic amino acid transporter-1, CCK: Cholecystokinin.
